# Bis{2-eth­oxy-6-[2-(methyl­ammonio)ethyl­imino­meth­yl]phenolato}thio­cyanato­zinc(II) nitrate

**DOI:** 10.1107/S1600536810000036

**Published:** 2010-01-09

**Authors:** Chen-Yi Wang, Zhi-Ping Han, Xiang Wu, Cai-Jun Yuan, Jun-Bo Zhou

**Affiliations:** a Department of Chemistry, Huzhou University, Huzhou 313000, People’s Republic of China

## Abstract

In the title compound, [Zn(NCS)(C_12_H_18_N_2_O_2_)_2_]NO_3_, the Zn^II^ ion is chelated by the phenolate O and imine N atoms from two zwitterionic Schiff base ligands and is also coordinated by the N atom of a thio­cyanate ligand, giving a distorted trigonal-bipyramidal geometry. Intra­molecular N—H⋯O hydrogen bonds are observed in the complex cation. The nitrate anions are linked to the complex cations through N—H⋯O hydrogen bonds.

## Related literature

For related structures, see: Zhang & Wang (2007 ▶); Adams *et al.* (2003 ▶).
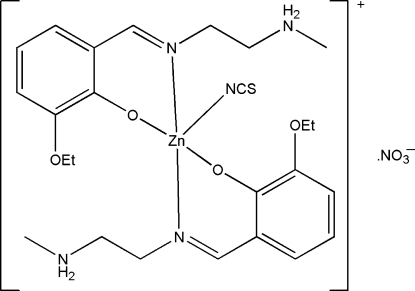


## Experimental

### 

#### Crystal data

[Zn(NCS)(C_12_H_18_N_2_O_2_)_2_]NO_3_
*M* *_r_* = 630.03
Monoclinic, 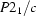

*a* = 10.601 (2) Å
*b* = 23.335 (3) Å
*c* = 13.749 (2) Å
β = 112.218 (3)°
*V* = 3148.6 (9) Å^3^
*Z* = 4
Mo *K*α radiation
μ = 0.90 mm^−1^
*T* = 298 K
0.20 × 0.20 × 0.18 mm

#### Data collection

Bruker SMART CCD area-detector diffractometer
Absorption correction: multi-scan (*SADABS*; Sheldrick, 1996 ▶) *T*_min_ = 0.841, *T*_max_ = 0.856
18443 measured reflections
6818 independent reflections
3644 reflections with *I* > 2σ(*I*)
*R*_int_ = 0.139

#### Refinement

*R*[*F* ^2^ > 2σ(*F* ^2^)] = 0.059
*wR*(*F* ^2^) = 0.157
*S* = 0.91
6818 reflections
365 parameters
6 restraints
H-atom parameters constrained
Δρ_max_ = 0.69 e Å^−3^
Δρ_min_ = −0.63 e Å^−3^

### 

Data collection: *SMART* (Bruker, 1998 ▶); cell refinement: *SAINT* (Bruker, 1998 ▶); data reduction: *SAINT*; program(s) used to solve structure: *SHELXS97* (Sheldrick, 2008 ▶); program(s) used to refine structure: *SHELXL97* (Sheldrick, 2008 ▶); molecular graphics: *SHELXTL* (Sheldrick, 2008 ▶); software used to prepare material for publication: *SHELXTL*.

## Supplementary Material

Crystal structure: contains datablocks global, I. DOI: 10.1107/S1600536810000036/ci5009sup1.cif

Structure factors: contains datablocks I. DOI: 10.1107/S1600536810000036/ci5009Isup2.hkl

Additional supplementary materials:  crystallographic information; 3D view; checkCIF report

## Figures and Tables

**Table 1:** Selected bond lengths (Å)

Zn1—O3	1.985 (2)
Zn1—O1	1.999 (3)
Zn1—N6	2.056 (4)
Zn1—N1	2.100 (3)
Zn1—N3	2.104 (3)

**Table 2:** Hydrogen-bond geometry (Å, °)

*D*—H⋯*A*	*D*—H	H⋯*A*	*D*⋯*A*	*D*—H⋯*A*
N2—H2*B*⋯O3	0.90	1.96	2.750 (4)	145
N2—H2*B*⋯O4	0.90	2.39	3.078 (4)	133
N4—H4*B*⋯O1	0.90	1.85	2.697 (4)	157
N4—H4*B*⋯O2	0.90	2.42	3.027 (5)	125
N2—H2*A*⋯O7^i^	0.90	2.01	2.898 (5)	170
N2—H2*A*⋯O6^i^	0.90	2.52	3.183 (6)	131
N4—H4*A*⋯O5^ii^	0.90	2.03	2.894 (5)	160
N4—H4*A*⋯O7^ii^	0.90	2.31	3.066 (5)	141

Symmetry codes: (i) 
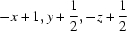
; (ii) 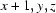.

**Fig. 1.**
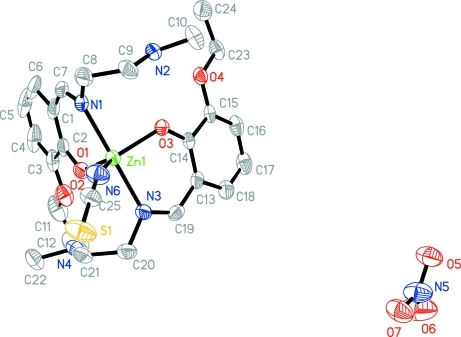
The asymmetric unit of the title compound, showing the atom-numbering scheme. Displacement ellipsoids are drawn at the 30% probability level. H atoms have been omitted for clarity.

## supplementary crystallographic information

### Comment

As part of our investigations into novel urease inhibitors, we have synthesized the title compound, a new Zn^II^ complex. The compound consists of a mononuclear zinc(II) complex cation and a nitrate anion. The Zn atom is chelated by the phenolate O and imine N atoms from two Schiff base ligands, and is coordinated by the N atom from a thiocyanate ligand, forming a trigonal-bipyramid geometry (Fig. 1). The coordinate bond lengths (Table 1) and angles are typical and are comparable with those observed in other similar zinc(II) complexes (Zhang & Wang, 2007; Adams *et al.*, 2003). The amine N atoms of the Schiff base ligands are protonated and take no part in the coordination to the Zn^II^ ion.

### Experimental

3-Ethoxysalicylaldehyde (0.2 mmol, 33.2 mg) and *N*-methylethane-1,2-diamine (0.2 mmol, 14.8 mg) were dissolved in MeOH (10 ml). The mixture was stirred at room temperature for 10 min to give a clear yellow solution. To this solution was added an aqueous solution (2 ml) of ammonium thiocyanate (0.2 mmol, 15.2 mg) and an aqueous solution (3 ml) of Zn(NO_3_)_2_.6H_2_O (0.1 mmol, 29.0 mg) with stirring. The resulting mixture was stirred for another 10 min at room temperature. After keeping the filtrate in air for a week, colourless block-shaped crystals were formed at the bottom of the vessel.

### Refinement

H atoms were placed in geometrically idealized positions and constrained to ride on their parent atoms, with C–H distances in the range 0.93–0.97 Å, N–H distances of 0.90 Å, and with *U*_iso_(H) set at 1.2*U*_eq_(C,*N*) and 1.5*U*_eq_(methyl C). During the refinement, the displacement parameters of atom O6 were restrained to an approximate isotropic behaviour. The unit cell contains four solvent accessible voids each with a volume of 53 Å^3^. But no significant electron density is found in these voids.

### Crystal data

**Table:**

[Zn(NCS)(C_12_H_18_N_2_O_2_)_2_]NO_3_	*F*(000) = 1320
*M**_r_* = 630.03	*D*_x_ = 1.329 Mg m^−^^3^
Monoclinic, *P*2_1_/*c*	Mo *K*α radiation, λ = 0.71073 Å
Hall symbol: -P 2ybc	Cell parameters from 3892 reflections
*a* = 10.601 (2) Å	θ = 2.3–25.5°
*b* = 23.335 (3) Å	µ = 0.90 mm^−^^1^
*c* = 13.749 (2) Å	*T* = 298 K
β = 112.218 (3)°	Block, colourless
*V* = 3148.6 (9) Å^3^	0.20 × 0.20 × 0.18 mm
*Z* = 4	

### Data collection

**Table:**

Bruker SMART CCD area-detector diffractometer	6818 independent reflections
Radiation source: fine-focus sealed tube	3644 reflections with *I* > 2σ(*I*)
graphite	*R*_int_ = 0.139
ω scan	θ_max_ = 27.0°, θ_min_ = 1.8°
Absorption correction: multi-scan (*SADABS*; Sheldrick, 1996)	*h* = −13→13
*T*_min_ = 0.841, *T*_max_ = 0.856	*k* = −29→28
18443 measured reflections	*l* = −17→15

### Refinement

**Table:**

Refinement on *F*^2^	Primary atom site location: structure-invariant direct methods
Least-squares matrix: full	Secondary atom site location: difference Fourier map
*R*[*F*^2^ > 2σ(*F*^2^)] = 0.059	Hydrogen site location: inferred from neighbouring sites
*wR*(*F*^2^) = 0.157	H-atom parameters constrained
*S* = 0.91	*w* = 1/[σ^2^(*F*_o_^2^) + (0.0647*P*)^2^] where *P* = (*F*_o_^2^ + 2*F*_c_^2^)/3
6818 reflections	(Δ/σ)_max_ = 0.001
365 parameters	Δρ_max_ = 0.69 e Å^−^^3^
6 restraints	Δρ_min_ = −0.63 e Å^−^^3^

### Special details

**Table:**

Geometry. All e.s.d.'s (except the e.s.d. in the dihedral angle between two l.s. planes) are estimated using the full covariance matrix. The cell e.s.d.'s are taken into account individually in the estimation of e.s.d.'s in distances, angles and torsion angles; correlations between e.s.d.'s in cell parameters are only used when they are defined by crystal symmetry. An approximate (isotropic) treatment of cell e.s.d.'s is used for estimating e.s.d.'s involving l.s. planes.
Refinement. Refinement of *F*^2^ against ALL reflections. The weighted *R*-factor *wR* and goodness of fit *S* are based on *F*^2^, conventional *R*-factors *R* are based on *F*, with *F* set to zero for negative *F*^2^. The threshold expression of *F*^2^ > σ(*F*^2^) is used only for calculating *R*-factors(gt) *etc*. and is not relevant to the choice of reflections for refinement. *R*-factors based on *F*^2^ are statistically about twice as large as those based on *F*, and *R*- factors based on ALL data will be even larger.

### Fractional atomic coordinates and isotropic or equivalent isotropic displacement parameters (Å^2^)

**Table:**

	*x*	*y*	*z*	*U*_iso_*/*U*_eq_	
Zn1	0.89814 (4)	0.910567 (18)	0.15980 (3)	0.04957 (18)	
N1	0.9386 (4)	0.99186 (14)	0.1129 (3)	0.0615 (9)	
N2	0.7214 (3)	1.05264 (13)	0.1565 (2)	0.0540 (8)	
H2A	0.7686	1.0855	0.1636	0.065*	
H2B	0.7705	1.0288	0.2085	0.065*	
N3	0.8674 (3)	0.82940 (12)	0.2138 (3)	0.0520 (8)	
N4	1.1307 (3)	0.78461 (14)	0.2169 (3)	0.0644 (9)	
H4A	1.1340	0.7610	0.2695	0.077*	
H4B	1.1352	0.8208	0.2404	0.077*	
N5	0.1534 (6)	0.6837 (2)	0.3942 (4)	0.1041 (16)	
N6	0.7831 (4)	0.88758 (17)	0.0075 (3)	0.0770 (11)	
O1	1.0989 (3)	0.89846 (11)	0.2324 (2)	0.0599 (7)	
O2	1.3351 (3)	0.86419 (17)	0.3710 (3)	0.0830 (10)	
O3	0.8145 (3)	0.94858 (10)	0.24990 (18)	0.0515 (6)	
O4	0.8062 (3)	1.02305 (12)	0.3908 (2)	0.0574 (7)	
O5	0.1139 (4)	0.73228 (16)	0.4024 (3)	0.1062 (12)	
O6	0.2187 (7)	0.6552 (2)	0.4726 (4)	0.175 (2)	
O7	0.1510 (4)	0.66460 (16)	0.3124 (3)	0.1077 (13)	
S1	0.65759 (17)	0.81681 (6)	−0.16349 (11)	0.1041 (5)	
C1	1.1809 (5)	0.9943 (2)	0.2309 (4)	0.0709 (13)	
C2	1.1969 (4)	0.9368 (2)	0.2642 (3)	0.0609 (11)	
C3	1.3288 (5)	0.9196 (3)	0.3372 (4)	0.0748 (14)	
C4	1.4340 (6)	0.9584 (3)	0.3697 (5)	0.106 (2)	
H4	1.5196	0.9468	0.4160	0.128*	
C5	1.4143 (8)	1.0148 (4)	0.3344 (5)	0.124 (3)	
H5	1.4868	1.0405	0.3576	0.149*	
C6	1.2928 (7)	1.0325 (3)	0.2677 (4)	0.0959 (19)	
H6	1.2814	1.0704	0.2451	0.115*	
C7	1.0547 (6)	1.01704 (19)	0.1556 (4)	0.0741 (14)	
H7	1.0579	1.0549	0.1353	0.089*	
C8	0.8286 (5)	1.0229 (2)	0.0317 (4)	0.0824 (15)	
H8A	0.8086	1.0040	−0.0353	0.099*	
H8B	0.8593	1.0615	0.0261	0.099*	
C9	0.7009 (5)	1.02618 (19)	0.0535 (3)	0.0665 (12)	
H9A	0.6336	1.0483	−0.0017	0.080*	
H9B	0.6648	0.9878	0.0516	0.080*	
C10	0.5889 (4)	1.0648 (2)	0.1656 (4)	0.0857 (15)	
H10A	0.5390	1.0922	0.1130	0.128*	
H10B	0.6047	1.0801	0.2341	0.128*	
H10C	0.5372	1.0300	0.1557	0.128*	
C11	1.4620 (5)	0.8444 (3)	0.4510 (5)	0.116 (2)	
H11A	1.4898	0.8705	0.5102	0.139*	
H11B	1.5329	0.8439	0.4225	0.139*	
C12	1.4452 (7)	0.7866 (3)	0.4864 (6)	0.146 (3)	
H12A	1.3661	0.7858	0.5045	0.219*	
H12B	1.5243	0.7767	0.5468	0.219*	
H12C	1.4340	0.7597	0.4310	0.219*	
C13	0.8233 (3)	0.86814 (17)	0.3627 (3)	0.0508 (9)	
C14	0.8163 (3)	0.92729 (16)	0.3392 (3)	0.0464 (9)	
C15	0.8116 (3)	0.96620 (18)	0.4179 (3)	0.0505 (10)	
C16	0.8127 (4)	0.9463 (2)	0.5121 (3)	0.0671 (12)	
H16	0.8114	0.9721	0.5632	0.081*	
C17	0.8156 (5)	0.8877 (2)	0.5319 (4)	0.0801 (14)	
H17	0.8150	0.8747	0.5957	0.096*	
C18	0.8192 (4)	0.8498 (2)	0.4591 (4)	0.0695 (12)	
H18	0.8190	0.8108	0.4728	0.083*	
C19	0.8394 (4)	0.82363 (17)	0.2956 (3)	0.0559 (10)	
H19	0.8281	0.7862	0.3143	0.067*	
C20	0.8790 (4)	0.77626 (17)	0.1597 (4)	0.0689 (12)	
H20A	0.7965	0.7712	0.0977	0.083*	
H20B	0.8866	0.7439	0.2059	0.083*	
C21	0.9995 (4)	0.77629 (18)	0.1273 (3)	0.0670 (12)	
H21A	1.0022	0.7402	0.0933	0.080*	
H21B	0.9881	0.8066	0.0764	0.080*	
C22	1.2507 (5)	0.7733 (2)	0.1879 (4)	0.0896 (15)	
H22A	1.2468	0.7346	0.1632	0.134*	
H22B	1.3331	0.7787	0.2484	0.134*	
H22C	1.2494	0.7993	0.1334	0.134*	
C23	0.8179 (4)	1.0643 (2)	0.4712 (3)	0.0668 (12)	
H23A	0.9029	1.0586	0.5306	0.080*	
H23B	0.7435	1.0594	0.4952	0.080*	
C24	0.8139 (5)	1.1231 (2)	0.4286 (4)	0.0909 (16)	
H24A	0.8793	1.1261	0.3960	0.136*	
H24B	0.8354	1.1504	0.4847	0.136*	
H24C	0.7244	1.1308	0.3775	0.136*	
C25	0.7319 (5)	0.85823 (19)	−0.0634 (4)	0.0663 (12)	

### Atomic displacement parameters (Å^2^)

**Table:**

	*U*^11^	*U*^22^	*U*^33^	*U*^12^	*U*^13^	*U*^23^
Zn1	0.0578 (3)	0.0483 (3)	0.0503 (3)	−0.0002 (2)	0.0291 (2)	−0.0045 (2)
N1	0.088 (3)	0.054 (2)	0.067 (2)	0.003 (2)	0.057 (2)	0.0022 (18)
N2	0.061 (2)	0.0538 (19)	0.0544 (19)	0.0034 (16)	0.0300 (17)	−0.0049 (16)
N3	0.0515 (19)	0.0462 (18)	0.060 (2)	−0.0018 (15)	0.0231 (17)	−0.0066 (16)
N4	0.074 (2)	0.057 (2)	0.065 (2)	0.0096 (18)	0.029 (2)	−0.0055 (18)
N5	0.161 (5)	0.073 (3)	0.075 (3)	0.019 (3)	0.040 (3)	0.017 (3)
N6	0.094 (3)	0.071 (2)	0.058 (2)	0.009 (2)	0.020 (2)	−0.007 (2)
O1	0.0493 (16)	0.0565 (16)	0.0770 (19)	−0.0063 (12)	0.0276 (15)	−0.0131 (14)
O2	0.0466 (18)	0.117 (3)	0.082 (2)	0.0016 (18)	0.0202 (17)	−0.016 (2)
O3	0.0676 (17)	0.0498 (15)	0.0483 (14)	0.0008 (13)	0.0346 (13)	0.0014 (12)
O4	0.0657 (18)	0.0639 (18)	0.0540 (16)	−0.0044 (14)	0.0355 (14)	−0.0120 (14)
O5	0.152 (3)	0.074 (2)	0.105 (3)	0.027 (2)	0.063 (3)	0.007 (2)
O6	0.280 (5)	0.103 (3)	0.127 (3)	0.022 (3)	0.057 (3)	0.006 (3)
O7	0.158 (4)	0.098 (3)	0.081 (2)	0.029 (2)	0.061 (3)	0.000 (2)
S1	0.1313 (13)	0.0791 (9)	0.0851 (9)	0.0145 (8)	0.0218 (9)	−0.0306 (8)
C1	0.093 (4)	0.082 (3)	0.064 (3)	−0.037 (3)	0.059 (3)	−0.028 (3)
C2	0.064 (3)	0.074 (3)	0.063 (3)	−0.021 (2)	0.046 (2)	−0.025 (2)
C3	0.057 (3)	0.114 (4)	0.068 (3)	−0.027 (3)	0.040 (3)	−0.031 (3)
C4	0.076 (4)	0.180 (7)	0.077 (4)	−0.052 (4)	0.046 (3)	−0.033 (4)
C5	0.124 (6)	0.189 (8)	0.086 (4)	−0.103 (6)	0.069 (4)	−0.050 (5)
C6	0.131 (5)	0.108 (4)	0.081 (4)	−0.071 (4)	0.075 (4)	−0.031 (3)
C7	0.123 (4)	0.052 (3)	0.086 (3)	−0.013 (3)	0.084 (4)	−0.010 (3)
C8	0.125 (4)	0.071 (3)	0.077 (3)	0.025 (3)	0.067 (3)	0.017 (3)
C9	0.084 (3)	0.067 (3)	0.052 (2)	0.016 (2)	0.030 (2)	−0.007 (2)
C10	0.068 (3)	0.116 (4)	0.081 (3)	0.021 (3)	0.037 (3)	−0.012 (3)
C11	0.057 (3)	0.181 (7)	0.100 (4)	0.009 (4)	0.018 (3)	−0.027 (5)
C12	0.107 (5)	0.160 (7)	0.141 (6)	0.053 (5)	0.012 (5)	0.010 (6)
C13	0.038 (2)	0.064 (3)	0.054 (2)	0.0025 (18)	0.0217 (18)	0.010 (2)
C14	0.0313 (19)	0.062 (2)	0.050 (2)	−0.0002 (17)	0.0201 (17)	−0.0017 (19)
C15	0.036 (2)	0.073 (3)	0.049 (2)	0.0034 (18)	0.0226 (18)	−0.001 (2)
C16	0.059 (3)	0.102 (4)	0.046 (2)	0.011 (2)	0.026 (2)	0.002 (2)
C17	0.078 (3)	0.117 (4)	0.057 (3)	0.028 (3)	0.038 (3)	0.030 (3)
C18	0.064 (3)	0.080 (3)	0.072 (3)	0.021 (2)	0.034 (2)	0.030 (3)
C19	0.046 (2)	0.050 (2)	0.069 (3)	−0.0051 (18)	0.020 (2)	0.009 (2)
C20	0.076 (3)	0.047 (2)	0.084 (3)	−0.008 (2)	0.030 (3)	−0.014 (2)
C21	0.079 (3)	0.052 (2)	0.072 (3)	0.003 (2)	0.031 (3)	−0.020 (2)
C22	0.084 (3)	0.098 (4)	0.099 (4)	0.014 (3)	0.049 (3)	−0.020 (3)
C23	0.053 (3)	0.089 (3)	0.060 (3)	−0.002 (2)	0.023 (2)	−0.026 (3)
C24	0.104 (4)	0.082 (4)	0.105 (4)	−0.024 (3)	0.060 (3)	−0.040 (3)
C25	0.078 (3)	0.061 (3)	0.059 (3)	0.018 (2)	0.024 (2)	0.001 (2)

### Geometric parameters (Å, °)

**Table:**

Zn1—O3	1.985 (2)	C8—C9	1.495 (6)
Zn1—O1	1.999 (3)	C8—H8A	0.97
Zn1—N6	2.056 (4)	C8—H8B	0.97
Zn1—N1	2.100 (3)	C9—H9A	0.97
Zn1—N3	2.104 (3)	C9—H9B	0.97
N1—C7	1.288 (6)	C10—H10A	0.96
N1—C8	1.465 (5)	C10—H10B	0.96
N2—C9	1.484 (5)	C10—H10C	0.96
N2—C10	1.485 (5)	C11—C12	1.466 (8)
N2—H2A	0.90	C11—H11A	0.97
N2—H2B	0.90	C11—H11B	0.97
N3—C19	1.274 (5)	C12—H12A	0.96
N3—C20	1.475 (5)	C12—H12B	0.96
N4—C21	1.481 (5)	C12—H12C	0.96
N4—C22	1.494 (5)	C13—C18	1.410 (6)
N4—H4A	0.90	C13—C14	1.413 (5)
N4—H4B	0.90	C13—C19	1.442 (5)
N5—O7	1.201 (5)	C14—C15	1.428 (5)
N5—O5	1.229 (5)	C15—C16	1.373 (5)
N5—O6	1.232 (6)	C16—C17	1.392 (6)
N6—C25	1.147 (5)	C16—H16	0.93
O1—C2	1.315 (4)	C17—C18	1.347 (6)
O2—C3	1.368 (6)	C17—H17	0.93
O2—C11	1.454 (6)	C18—H18	0.93
O3—C14	1.318 (4)	C19—H19	0.93
O4—C15	1.373 (5)	C20—C21	1.504 (6)
O4—C23	1.435 (4)	C20—H20A	0.97
S1—C25	1.621 (5)	C20—H20B	0.97
C1—C2	1.407 (6)	C21—H21A	0.97
C1—C6	1.416 (6)	C21—H21B	0.97
C1—C7	1.448 (7)	C22—H22A	0.96
C2—C3	1.435 (6)	C22—H22B	0.96
C3—C4	1.373 (7)	C22—H22C	0.96
C4—C5	1.392 (9)	C23—C24	1.486 (6)
C4—H4	0.93	C23—H23A	0.97
C5—C6	1.334 (9)	C23—H23B	0.97
C5—H5	0.93	C24—H24A	0.96
C6—H6	0.93	C24—H24B	0.96
C7—H7	0.93	C24—H24C	0.96
			
O3—Zn1—O1	113.20 (11)	N2—C10—H10B	109.5
O3—Zn1—N6	121.28 (14)	H10A—C10—H10B	109.5
O1—Zn1—N6	125.52 (14)	N2—C10—H10C	109.5
O3—Zn1—N1	88.83 (11)	H10A—C10—H10C	109.5
O1—Zn1—N1	88.76 (13)	H10B—C10—H10C	109.5
N6—Zn1—N1	91.96 (15)	O2—C11—C12	110.4 (5)
O3—Zn1—N3	90.95 (11)	O2—C11—H11A	109.6
O1—Zn1—N3	88.52 (11)	C12—C11—H11A	109.6
N6—Zn1—N3	90.76 (14)	O2—C11—H11B	109.6
N1—Zn1—N3	176.95 (14)	C12—C11—H11B	109.6
C7—N1—C8	118.1 (4)	H11A—C11—H11B	108.1
C7—N1—Zn1	122.9 (3)	C11—C12—H12A	109.5
C8—N1—Zn1	119.0 (3)	C11—C12—H12B	109.5
C9—N2—C10	111.0 (3)	H12A—C12—H12B	109.5
C9—N2—H2A	109.4	C11—C12—H12C	109.5
C10—N2—H2A	109.4	H12A—C12—H12C	109.5
C9—N2—H2B	109.4	H12B—C12—H12C	109.5
C10—N2—H2B	109.4	C18—C13—C14	119.6 (4)
H2A—N2—H2B	108.0	C18—C13—C19	115.9 (4)
C19—N3—C20	116.6 (3)	C14—C13—C19	124.5 (4)
C19—N3—Zn1	121.7 (3)	O3—C14—C13	124.2 (3)
C20—N3—Zn1	121.7 (3)	O3—C14—C15	118.3 (3)
C21—N4—C22	112.4 (3)	C13—C14—C15	117.5 (4)
C21—N4—H4A	109.1	C16—C15—O4	124.6 (4)
C22—N4—H4A	109.1	C16—C15—C14	120.6 (4)
C21—N4—H4B	109.1	O4—C15—C14	114.8 (3)
C22—N4—H4B	109.1	C15—C16—C17	120.6 (4)
H4A—N4—H4B	107.9	C15—C16—H16	119.7
O7—N5—O5	122.7 (5)	C17—C16—H16	119.7
O7—N5—O6	115.1 (5)	C18—C17—C16	120.3 (4)
O5—N5—O6	121.1 (5)	C18—C17—H17	119.9
C25—N6—Zn1	158.4 (4)	C16—C17—H17	119.9
C2—O1—Zn1	128.9 (3)	C17—C18—C13	121.3 (4)
C3—O2—C11	118.1 (4)	C17—C18—H18	119.4
C14—O3—Zn1	124.1 (2)	C13—C18—H18	119.4
C15—O4—C23	117.2 (3)	N3—C19—C13	127.7 (4)
C2—C1—C6	120.2 (5)	N3—C19—H19	116.1
C2—C1—C7	123.2 (4)	C13—C19—H19	116.1
C6—C1—C7	116.6 (5)	N3—C20—C21	113.0 (3)
O1—C2—C1	123.9 (4)	N3—C20—H20A	109.0
O1—C2—C3	118.8 (4)	C21—C20—H20A	109.0
C1—C2—C3	117.3 (4)	N3—C20—H20B	109.0
O2—C3—C4	125.6 (6)	C21—C20—H20B	109.0
O2—C3—C2	114.3 (4)	H20A—C20—H20B	107.8
C4—C3—C2	120.1 (6)	N4—C21—C20	112.9 (4)
C3—C4—C5	120.9 (6)	N4—C21—H21A	109.0
C3—C4—H4	119.6	C20—C21—H21A	109.0
C5—C4—H4	119.6	N4—C21—H21B	109.0
C6—C5—C4	120.8 (6)	C20—C21—H21B	109.0
C6—C5—H5	119.6	H21A—C21—H21B	107.8
C4—C5—H5	119.6	N4—C22—H22A	109.5
C5—C6—C1	120.8 (6)	N4—C22—H22B	109.5
C5—C6—H6	119.6	H22A—C22—H22B	109.5
C1—C6—H6	119.6	N4—C22—H22C	109.5
N1—C7—C1	128.6 (4)	H22A—C22—H22C	109.5
N1—C7—H7	115.7	H22B—C22—H22C	109.5
C1—C7—H7	115.7	O4—C23—C24	109.5 (3)
N1—C8—C9	113.2 (4)	O4—C23—H23A	109.8
N1—C8—H8A	108.9	C24—C23—H23A	109.8
C9—C8—H8A	108.9	O4—C23—H23B	109.8
N1—C8—H8B	108.9	C24—C23—H23B	109.8
C9—C8—H8B	108.9	H23A—C23—H23B	108.2
H8A—C8—H8B	107.8	C23—C24—H24A	109.5
N2—C9—C8	113.3 (4)	C23—C24—H24B	109.5
N2—C9—H9A	108.9	H24A—C24—H24B	109.5
C8—C9—H9A	108.9	C23—C24—H24C	109.5
N2—C9—H9B	108.9	H24A—C24—H24C	109.5
C8—C9—H9B	108.9	H24B—C24—H24C	109.5
H9A—C9—H9B	107.7	N6—C25—S1	179.2 (5)
N2—C10—H10A	109.5		

### Hydrogen-bond geometry (Å, °)

**Table:**

*D*—H···*A*	*D*—H	H···*A*	*D*···*A*	*D*—H···*A*
N2—H2B···O3	0.90	1.96	2.750 (4)	145
N2—H2B···O4	0.90	2.39	3.078 (4)	133
N4—H4B···O1	0.90	1.85	2.697 (4)	157
N4—H4B···O2	0.90	2.42	3.027 (5)	125
N2—H2A···O7^i^	0.90	2.01	2.898 (5)	170
N2—H2A···O6^i^	0.90	2.52	3.183 (6)	131
N4—H4A···O5^ii^	0.90	2.03	2.894 (5)	160
N4—H4A···O7^ii^	0.90	2.31	3.066 (5)	141

Symmetry codes: (i) −*x*+1, *y*+1/2, −*z*+1/2; (ii) *x*+1, *y*, *z*.
